# The effectiveness of water jet flossing and interdental flossing for oral hygiene in orthodontic patients with fixed appliances: a randomized clinical trial

**DOI:** 10.1186/s12903-024-04166-0

**Published:** 2024-04-27

**Authors:** Hani S. AlMoharib, Afyaa Alqasem, Ghaida Almusfer, Mohammad A. Aldosari, Hossam Waleed Almadhoon

**Affiliations:** 1 Periodontics and Community Dentistry, College of Dentistry, King Saud University, Riyadh, Saudi Arabia; 2 College of Dentistry, King Saud University, Riyadh, Saudi Arabia; 3 Orthodontics and Pediatric Dentistry, College of Dentistry, King Saud University, Riyadh, Saudi Arabia; 4 Faculty of Dentistry, Al-Azhar University-Gaza, Gaza Strip, Palestine

**Keywords:** Water jet, Dental floss, Orthodontic treatment, Plaque index, Gingivitis

## Abstract

**Background:**

Orthodontic treatment presents challenges with plaque accumulation around brackets, archwires, and elastics, leading to retained plaque and gingival inflammation. Conventional toothbrushing may not be enough, requiring additional oral hygiene aids like interproximal brushes, dental flosses, and water flossers. Limited research exists on comparing water flossing and interdental flossing in orthodontic patients. Therefore, this study aims to assess their effectiveness in maintaining oral hygiene during active orthodontic treatment.

**Methods:**

A single-blind, randomized, parallel clinical study recruited orthodontic patients with full-mouth brackets and archwires. Thirty participants were randomly assigned to either water jet flossing or interdental flossing groups. All participants were instructed to brush twice daily with a provided toothbrush and toothpaste and use the assigned intervention once daily at night. Clinical measures, including the Gingival Bleeding Index (BI), Plaque Index (PI), and Gingival Index (GI), were recorded at baseline and day 14. Descriptive statistics and statistical tests were performed using SPSS software.

**Results:**

The water jet flossing group demonstrated a slightly higher, albeit non-significant, benefit in plaque removal (median difference of 6.79%%, *P* = 0.279) and bleeding reduction (median difference of 5.21%%, *P* = 0.172) compared to the interdental flossing group after two weeks. Both groups showed significant reductions in gingival bleeding index and plaque index from baseline to the 2-week follow-up. The interdental flossing group had median mean percentage differences of 16.13%% (plaque index) and 23.57% (gingival bleeding index), while the water jet flossing group had median percentage differences of 21.87% (plaque index) and 32.29% (gingival bleeding index). No significant changes in gingival index grades were observed in either group.

**Conclusion:**

Both water jet flossing and interdental flossing were effective in reducing plaque accumulation and gingival bleeding among orthodontic patients. While no significant differences were found between the two methods, water jet flossing showed a potential advantage. Further research is needed to validate its effectiveness, assess long-term impact, and understand its benefits for orthodontic patients.

## Introduction

Plaque accumulation poses a significant challenge for orthodontic patients, as the presence of brackets, archwires, and elastics can make it difficult to clean all tooth surfaces effectively [1]. This can result in the retention of plaque and subsequent gingival inflammation [2]. Multiple studies have reported that the use of multibracket appliances can exacerbate both plaque and bleeding indexes in orthodontic patients along with the presence of active biofilm leading to the formation of white spots on the enamel [3–5, 6, 7, 8, 9, 10, 11]. Additionally, the literature has documented various adverse effects associated with orthodontic treatment, such as gingival enlargement, tooth decalcification, and soft tissue recession [3, 12–14, 7]. Along with changes in the distribution of dental biofilm, characterized by change in the location of plaque and an Increase of supragingival plaque in the interdental area and on the vestibular surface of the teeth, and quantitative changes in plaque are observed after just one week after starting the orthodontic treatment and become more consistent three months later [15, 16].

Effective mechanical plaque removal is essential for maintaining good oral hygiene in patients with fixed orthodontic appliances [17, 18–20, 21, 22, 23, 24, 25, 26]. However, conventional toothbrushing alone may not be sufficient for orthodontic patients due to an increase in plaque retention and limited accessibility [27]. To address this issue, several oral hygiene aids have been developed to help facilitate oral hygiene maintenance in orthodontic patients in addition to manual toothbrushing without affecting the orthodontic appliance negatively [28, 19]. These aids include interproximal brushes, dental flosses, and water flossers [27–29]. Previous studies have suggested that approximately 20-40% of patients with fixed orthodontic appliances may have suboptimal plaque control when using manual toothbrushes alone [30, 31].

The use of a water flosser has been reported to aid in the reduction of gingival inflammation and pathogenic bacteria in patients with implants, crowns, bridges, and diabetes [1, 32, 33, 34]. Water flossers are particularly useful for eliminating debris from inaccessible areas around orthodontic appliances [35]. Furthermore, studies have shown that using a water flosser as an adjunct to manual tooth brushing can lead to superior improvement in periodontal health compared to using only one device [36]. On the other hand, other studies have documented that daily interdental flossing alone can decrease bleeding, plaque, and inflammation indexes [37–39]. A study by Barnes et al. [40, 41] on non-orthodontic adults demonstrated that both water flossing and interdental flossing can equally reduce gingivitis and gingival bleeding over the same period. Furthermore, water flossers have been recommended to help orthodontic patients [42, 43] along with regular visits to the dental hygienist [44].

The available literature on the comparison of water flossing and interdental flossing in patients with fixed orthodontic appliances is limited, with only a few published randomised clinical trials [1, 29]. As a result, there is insufficient evidence to determine the effectiveness of dental water flossers compared to interdental flossing for maintaining oral hygiene in orthodontic patients [45]. This study aims to compare the effectiveness of water jet flossing with interdental flossing in maintaining oral hygiene in patients currently undergoing active orthodontic treatment with fixed appliances.

## Methodology

The IRB is registered with the Office for Human Research Protection (OHRP) with OHRP Institution Registration No.: IORG0006829 and the trial was approved by TCTR Committee on 26/9/2023. The TCTR identification number is TCTR20230926005.

### Study design

This was a single-blind, randomised, parallel clinical study that involved patients currently undergoing active orthodontic treatment with full-mouth brackets and archwires. Participants were randomly assigned to one of two groups based on the method of intervention:

(1) Water jet flossing in conjunction with a manual toothbrush and fluoridated toothpaste.

(2) Interdental flossing in conjunction with a manual toothbrush and fluoridated toothpaste.

The blinding in this study was implemented at the clinician level, meaning that the clinician who assessed the outcomes and recorded the data was unaware of which intervention each participant received.

### Target population and sample size

Participants were recruited from the Orthodontic Clinics at King Saud University in Riyadh, Saudi Arabia. Prior to the commencement of the study, all participants willingly provided informed consent. The sample size for the study was determined using the formula n = [(Zα/2 + Zβ)^2^ × {2(Sd)^2^}]/ (_∆_)^2^, where ∆ is the mean difference between two groups and Sd is the standard deviation( Mean bleeding index difference of 25 and standard deviation of 22.79), with 80% power, at 5% alpha level Based on this calculation, a sample size of 15 subjects in each group and total of 30 participants was determined.

### Eligibility criteria for participant selection

#### Inclusion criteria

Participants were included in the study if they met all of the following criteria:

(1) Physically healthy without any medical conditions.

(2) Aged 18 years or older.

(3) Have natural dentition with at least 24 teeth in the mouth, excluding the third molars.

(4) Have less than 5 mm of anterior crowding or spacing, with adequate overjet and overbite.

(5) Have the physical and mental ability to maintain adequate oral hygiene.

(6) Have good periodontal health without an immediate need for any dental procedure.

#### Exclusion criteria

Participants were excluded from the study if they had any of the following:

(1) A medical history of immunologic and inflammatory diseases, liver diseases, diabetes mellitus, current pregnancy, or any medical condition that limits manual dexterity.

(2) A recent history of medications that may alter gingival health, such as anti-sialagogues, steroids, and antibiotics.

(3) Active periodontal disease.

(4) Removable orthodontic appliances.

(5) Not using any type of mouthwash.

### Study procedure

**Fig. 1:**
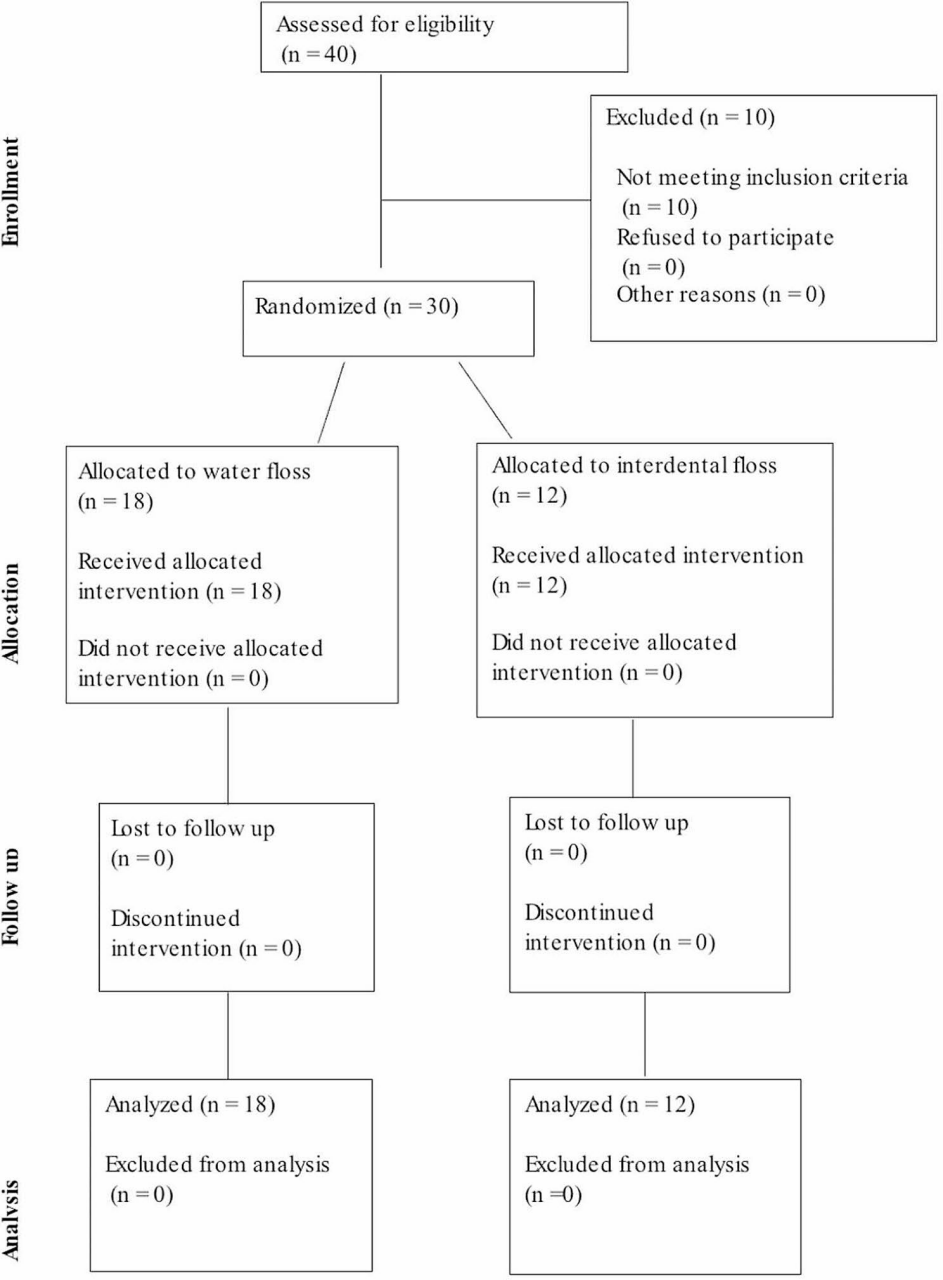
CONSORT flow diagram

The trial was conducted followed the (CONSORT) guidelines (Fig. 1). At the beginning of the study, all participants were provided with a soft manual toothbrush and fluoridated toothpaste. Participants were then assigned to either a water flosser group or an interdental flossing group using simple randomization. They were instructed to use only the assigned product and not to use any other aids, such as mouth rinses or toothpicks. Verbal, visual, and written instructions were given to all participants regarding the proper use of their assigned intervention at the baseline visit and again on day 14. Additionally, participants were instructed to brush with the provided toothpaste and manual brush for two minutes twice daily (in the morning and at night) and to use the assigned intervention once a day at night (either the water flosser or interdental floss). It was ensured that the participants used the water flosser or interdental floss in a manner that did not affect the integrity of the fixed orthodontic devices and did not harm the oral tissues. Detailed instructions were provided to participants on the proper technique of using the assigned intervention, emphasizing the importance of gentle and careful application to avoid any adverse effects on the orthodontic appliances or oral tissues.

### Clinical measures

Each participant was assigned to the same clinician at both the baseline and day 14 assessments. The following clinical measures were recorded for the assessment of periodontal health status in six selected teeth (16, 12, 24, 36, 32, and 44) using the FDI teeth numbering system:

1. Gingival Bleeding Index (BI) [29]: This index assesses all four surfaces of teeth to determine if probing with a probe will result in bleeding or not. The presence of bleeding was recorded as (+), and the absence of bleeding was recorded as (−).
2. Plaque Index (PI) [46]: This index records the presence or absence of spuragingival plaque on all four tooth surfaces. The presence of plaque was recorded as (+), and the absence of plaque was recorded as (−).
3. Gingival Index (GI) [47]: This index grades gingival inflammation. Grade (0) indicates normal gingiva with no inflammation or bleeding, Grade (1) indicates mild inflammation and erythema with no bleeding, Grade (2) indicates bleeding on probing and erythema, and Grade (3) indicates severe inflammation with swelling, erythema, and tendency to spontaneous bleeding.

These examinations were conducted using a periodontal examination kit and plaque disclosing agent. Each participant was provided with a consent form that included a description of the study, its objective, and their role in it.

### Statistical analysis

The statistical analysis was conducted using SPSS (Statistical Package for Social Sciences) version 26.0 software. Descriptive statistics, such as median, inter-quartile range(IQR), frequencies, and percentages, were employed to provide a comprehensive overview of the skewed quantitative and categorical outcome variables. To examine the differences between the initial and follow-up stages of the study, as well as between the interdental and water jet flossing groups, appropriate statistical tests were employed. As the gingival bleeding index and plaque index values are not following normal distribution, the non-parametric tests (Mann-Whitney U-test and Wilcoxon sign rank test) were used to compare the mean ranks of percentage values between two groups and between initial & follow up stages in each of the two groups. Furthermore, Fisher’s exact test was used to compare the distribution of the gingival index between the two study group and between initial and follow up stages in each of the two groups. The statistical significance of the results was determined using a p-value threshold of ≤ 0.05.

## Results

A total of 30 participants were randomly assigned to two study groups: (1) conventional interdental flossing (12 participants) and (2) water jet flossing (18 participants). At baseline, the plaque index scores were similar between the interdental floss and water jet groups, with median percentages of 37.50% and 36.50%respectively. However, the water jet flossing group had a slightly higher gingival bleeding mean score than the interdental flossing group, with a mean difference of 5.61%, but the difference was not statistically significant (*P* = 0.518). Table 1 summarises the differences between both groups in terms of plaque and gingival bleeding indexes at baseline and after two weeks. The water jet flossing group provided a non-significantly higher plaque removal and bleeding reduction benefit compared to the interdental flossing group at two weeks, with between treatment mean differences of 3.12% and 8.39%, respectively (*P* = 0.279, *P* = 0.172, respectively).

Both interdental flossing and water jet flossing groups did not show significant changes in gingival index grades at baseline versus at two weeks. At baseline, the interdental flossing group had 83.3% grade 2 and 16.7% grade 3, while after two weeks, 91.7% had grade 2 and 8.3% had grade 3. In the water jet flossing group, initially, 94.4% had grade 2 and 5.6% had grade 3, and after two weeks, all participants had grade 2 gingival index (Table 1).

**Table 1:** Comparison of median percentage values of plaque index, gingival bleeding index & gingival index grades between two study groups of initial and follow up stages

Outcome variables	Study groups- Median % ( IQR)		Median% difference	p-value
	Interdental (*n* = 12)	Water jet flossing (*n* = 18)		
**Plaque Index**				
Initial stage	37.50(18.3)	36.45(40.0)		0.932*
Follow-up stage			6.79	
	21.37(19.6)	14.58(11.5)		0.279*
**Gingival bleeding Index**			3.51	
Initial stage		47.91(47.7)	5.21	0.518*
Follow- up stage	44.40(44.6)20.83(30.9)	15.62(13.5)		0.172*
	0.83(30.9)			
**Gingival Index grades**				
** Initial stage**				
2		10(83.3)	17(94.4)	0.347**
3		2(16.7)	1(5.6)	--
** Follow-up stage**				
2		11(91.7)	18(100)	0.400**
3		1(8.3)	0	--

*By using Mann-Whitney U-test;** By using Fisher’s exact test

Table 2 shows a statistically significant difference in the median percentage values of plaque and gingival bleeding indexes between the initial and follow-up stages in both study groups. Specifically, the median percentage values of gingival bleeding index and plaque index were significantly decreased from baseline to the 2-week follow-up in both the conventional interdental and water jet flossing groups. The median percentage difference of plaque index and gingival bleeding index values of the interdental group were 16.13% and 23.57%%, respectively, while for the water jet flossing group, they were 21.87%and 32.29%, respectively, which were higher than the values of the interdental flossing group.

**Table 2:** Comparison of median percentage values between initial and follow-up stages in both study groups

Study group and outcome variables	Stage – Mean % (Sd.,)		Median% difference		p-value*
	Initial	Follow-up			
**Interdental floss group**			16.13		
			23.57		
Plaque Index	37.50(18.3)	21.37(19.6)			0.010
	44.40(44.6)	20.83(30.9)			0.002
Gingival Bleeding Index					
	10 (83.3)	11 (91.7)			--
Gingival index grades	2 (16.7)	1 (8.3)			--
2					
3					
**Water jet flossing group**					
Plaque index	36.45(40.0)	14.58(11.5) 15.62(13.5)	21.87		< 0.0001
	47.91(47.7)	15.62(13.5)	32.29		
Gingival Bleeding index					< 0.0001
	17 (94.4)	18 (100)		--	
Gingival index grades	1 (5.6)	--	--	--	
2			--		--
3					--

* By using Wilcoxon Signed Rank test

## Discussion

Orthodontic patients often face challenges in maintaining proper oral hygiene due to the presence of brackets and archwires, which create additional spaces for plaque accumulation and other periodontal problems. The current study has evaluated two adjunctive methods to manual toothbrushes for maintaining oral hygiene in orthodontic patients undergoing treatment with fixed appliances: water jet flossing and conventional interdental flossing. Our findings indicate that both interdental cleaning methods demonstrate statistically significant differences in plaque and gingival bleeding scores between the groups in the 2nd week compared to the baseline, with no statistical difference between both methods.

Several studies have demonstrated the efficacy of water flosser and regular floss in reducing plaque accumulation, gingival inflammation, and bleeding across diverse populations [29, 48, 49]. A split-mouth randomised controlled clinical trial by Abdellatif et al. 2021 brought to light the significant reduction in plaque scores with the use of both regular floss and water flossers in patients with fair to poor oral hygiene [29]. A subsequent randomised controlled trial by Xu et al. (2023) found the additional use of water flossing alongside traditional tooth brushing to be more proficient in diminishing the gingival index and sulcus bleeding index among patients with gingivitis [48]. Corroborating these findings, Lyle et al.‘s (2020) study with 70 healthy participants noted a stronger reduction in plaque, gingival, and gingival bleeding indices when a water flosser was integrated with an electric toothbrush compared to using the toothbrush alone [49]. A synthesis of two randomised controlled trials conveyed that the superiority of a combination of manual tooth brushing and water flossing over exclusive manual tooth brushing or manual brushing with regular floss [40, 50]. Consistently, Goyal et al. (2018), in a comparative study between manual toothbrush plus water flosser to a manual toothbrush alone, demonstrated superior proficiency of the additive approach (manual toothbrush plus water flosser) in mitigating gingival bleeding and improving gingival health with a usage period of 4 weeks [36]. A systematic review by Worthington et al. (2019) further substantiated these claims, noting enhanced oral health outcomes in groups that supplemented tooth brushing with oral irrigation in contrast to those solely relying on tooth brushing [51].

In interpreting the results of our study, the prominent efficiency of water jet flossing in comparison to traditional interdental flossing to reduce plaque and gingival bleeding cannot be overlooked. These methods, when used alongside manual brushing, proved to make substantial improvements in oral hygiene, particularly potent in reinforcing orthodontic treatments [29, 48]. Our study showed marginally better performances in the water jet flossing group, albeit non-significant, over the span of two weeks, resulting in a plaque index score and a gingival index score of 21.87 and 32.29 respectively, compared to the conventional flossing group scores of 16.13 and 23.57. This subtle incline towards water jet flossing yields an intriguing space for further exploration.

Our findings harmoniously align with Sharma et al.‘s (2008) study, which illustrated a similar level of effectiveness from both techniques in reducing bleeding among adolescent patients with fixed orthodontic appliances [29]. They further found that water flossers surpass regular flossing and manual tooth brushing alone in depleting whole-mouth and interproximal plaque at two and four-week periods [29]. Nevertheless, dissenting from the trend, Mazzoleni et al. (2019) concluded in their randomised controlled trial that the dental water jet did not significantly enhance the home oral hygiene efficacy in orthodontic patients wearing a multi-bracket fixed appliance [1]. Taking a different approach, Sawan et al.‘s trial involving Saudi orthodontic patients compared super floss and water flosser, confirming that both methods engendered immediate amelioration in plaque index scores post-cleaning compared to pre-cleaning baseline. Interestingly, the study revealed a more substantial impact of water flosser on plaque removal, especially on the distal, interproximal surface of the molar tooth [52]. Caccianiga et al. (2022) further corroborated the argument of water flosser’s efficacy; they suggested that an oral irrigator in conjunction with a sonic toothbrush may balance oral hygiene in individuals with pathogenic flora, which subsequently reduces the risk of caries and gingivitis in orthodontic patients [53]. Observing these different study endpoints along with our current findings, the argument leans towards a broader application for water jet flossing, implying its potential benefits extending beyond simply orthodontic patients and reaching out to individuals with diverse periodontal conditions.

The potential benefits of water jet flossing can be traced back to its unique mechanism that serves a pulsating water stream [54]. This pulsation generates a hydrodynamic effect that dislodges and extricates plaque, debris, and food particles from the challenging interdental spaces and around orthodontic brackets [35, 55, 56]. Traditional flossing methods may struggle to navigate these intricate spaces, making the water jet flosser a particularly advantageous tool for orthodontic patients [54, 56]. In addition to effective cleaning, the pulsating action from water jet flossing has a massaging effect on the gingival tissues [54]. This massage may enhance circulation, diminish inflammation, and potentially contribute to the trend of lower gingival Index scores observed in the water jet flossing group [35, 55, 56]. On the contrary, interdental flossing relies on a mechanical process that scrapes the tooth surfaces to eradicate plaque [56]. While efficient, it lacks the intricate reach offered by water jet flossers. Apart from that, water jet flossing introduces a gentle, non-invasive cleaning approach, ideal for individuals wearing orthodontic appliances who might experience sensitivity or discomfort with traditional flossing [56]. The convenience, ease of use, and gentle touch of water jet flossers could better encourage orthodontic patients to comply with oral hygiene practices and therefore could foster improved periodontal health outcomes [56].

Our findings, when applied to a broader orthodontic population, are constrained by the limitation of a relatively small sample size. Furthermore, the two-week timeframe may have limited our ability to observe long-term effects of both flossing techniques on the overall oral health of orthodontic patients. Similarly, variations in personal levels of oral hygiene adherence among the patients may have affected flossing efficacy and outcomes. Despite certain limitations, our study followed a methodologically robust design, incorporating randomised assignment of techniques, which should limit potential bias and largely enhance the validity of our findings. However, expanding the research to longer follow-up periods and including more diversified groups with larger sample sizes would likely provide more insights into potential long-term benefits and variations among different oral hygiene maintenance strategies. Additionally, combining these approaches with ongoing dental professional care and monitoring would deliver personalised patient care maximising their oral health outcomes.

## Conclusion

Based on our findings, both water jet flossing and traditional interdental flossing serve as reliable and effective methods, significantly reducing plaque accumulation and gingival bleeding among orthodontic patients. Although the comparative analysis did not yield any statistically significant disparities in the effectiveness of the dental water jet and conventional dental floss, an observed marginal superiority of water jet flossing implies its potential to augment traditional flossing practices in orthodontic patient care. Future research is needed to further confirm the effectiveness of water jet flossing and to better understand its specific benefits for orthodontic patients, including its long-term impact on oral health, potential to enhance patient comfort during treatment, and its influence on patient compliance with oral hygiene practices.

## Data Availability

The datasets used and analyzed during the current study are available from the corresponding author on reasonable request.

## References

[1] Mazzoleni S, De Stefani A, Bordin C, Balasso P, Bruno G, Gracco A (2019). Dental water jet efficacy in the plaque control of orthodontic patients wearing fixed appliance: a randomized controlled trial. J Clin Experimental Dentistry.

[2] Ristic M, Svabic MV, Sasic M, Zelic O (2007). Clinical and microbiological effects of fixed orthodontic appliances on periodontal tissues in adolescents. Orthod Craniofac Res.

[3] Klukowska M, Bader A, Erbe C, Bellamy P, White DJ, Anastasia MK, Wehrbein H (2011). Plaque levels of patients with fixed orthodontic appliances measured by digital plaque image analysis. Am J Orthod Dentofac Orthop.

[4] Bollen A-M, Cunha-Cruz J, Bakko DW, Huang GJ, Hujoel PP (2008). The effects of orthodontic therapy on periodontal health: a systematic review of controlled evidence. J Am Dent Association.

[5] Atassi F, Awartani F (2010). Oral hygiene status among orthodontic patients. J Contemp Dent Pract.

[6] Francis PG, Parayaruthottam P, Antony V, Ummar F, Shaloob KMM, Hassan KJ (2019). A comparative assessment of the effect of professional oral hygiene measures on the periodontal health of patients undergoing fixed orthodontic appliance therapy. J Indian Orthodontic Soc.

[7] Kozak U, Sękowska A, Chałas R (2020). The effect of regime oral-hygiene intervention on the incidence of new white spot lesions in teenagers treated with fixed orthodontic appliances. Int J Environ Res Public Health.

[8] Thanetchaloempong W, Koontongkaew S, Utispan K (2022). Fixed orthodontic treatment increases cariogenicity and virulence gene expression in dental biofilm. J Clin Med.

[9] Mulimani P, Popowics T (2022). Effect of orthodontic appliances on the oral environment and microbiome. Front Dent Med.

[10] Skilbeck MG, Mei L, Mohammed H, Cannon RD, Farella M (2021). The effect of ligation methods on biofilm formation in patients undergoing multi-bracketed fixed orthodontic therapy—A systematic review. Orthod Craniofac Res.

[11] Skilbeck MG (2022). The effect of ligation methods on biofilm formation in patients undergoing multi-bracketed fixed orthodontic therapy–A systematic review. Orthod Craniofac Res.

[12] O’reilly M, Featherstone J (1987). Demineralization and remineralization around orthodontic appliances: an in vivo study. Am J Orthod Dentofac Orthop.

[13] Sim H-Y, Kim H-S, Jung D-U, Lee H, Lee J-W, Han K, Yun K-I (2017). Association between orthodontic treatment and periodontal diseases: results from a national survey. Angle Orthod.

[14] Christou V, Timmerman MF, Van der Velden U, Van der Weijden FA (1998). Comparison of different approaches of interdental oral hygiene: interdental brushes versus dental floss. J Periodontol.

[15] Kozak U, Lasota A, Chałas R (2021). Changes in distribution of dental biofilm after insertion of fixed orthodontic appliances. J Clin Med.

[16] Contaldo M, Lucchese A, Lajolo C, Rupe C, Di Stasio D, Romano A, Petruzzi M, Serpico R (2021). The oral microbiota changes in Orthodontic patients and effects on oral health: an overview. J Clin Med.

[17] Farook FF, Alrumi A, Aldalaan K, Ababneh K, Alshammari A, Al-Khamees AA, Albalawi F (2023). The efficacy of manual toothbrushes in patients with fixed orthodontic appliances: a randomized clinical trial. BMC Oral Health.

[18] Naqvi ZA, Almehrej BA, Alrabeeah MA, Alshahrani MA (2020). Comparison of manual orthodontic, powered and sonic tooth brushes in patients undergoing fixed orthodontics. Eur J Mol Clin Med.

[19] El Shehaby M, Mofti B, Montasser MA, Bearn D (2020). Powered vs manual tooth brushing in patients with fixed orthodontic appliances: a systematic review and meta-analysis. Am J Orthod Dentofac Orthop.

[20] Mylonopouloua M, Pepelassib E, Madianosb P, Halazonetisa DJ (2021). A randomized, 3-month, parallel-group clinical trial to compare the efficacy of electric 3-dimensional toothbrushes vs manual toothbrushes in maintaining oral health in patients with fixed orthodontic appliances. Am J Orthod Dentofac Orthop.

[21] Saengphen T, Koontongkaew S, Utispan K. Effectiveness of a combined toothbrushing technique on Cariogenic Dental Biofilm in Relation to Stainless Steel and Elastomeric Ligatures in Orthodontic patients: a Randomized Clinical Trial. Healthcare. Volume 11. No. 5. MDPI; 2023. 10.3390/healthcare11050731 PMC10000873 36900736

[22] Gomes LK, Sarmento CF, Seabra FR, Santos PB, Pinheiro FH. Randomized clinical controlled trial on the effectiveness ofconventional and orthodontic manual toothbrushes. Braz Oral Res. 2012:ul-Aug;26(4):360-5. 10.1590/s1806-83242012000400013. PMID: 22790501. 10.1590/s1806-83242012000400013 22790501

[23] Bhatia N, Ahluwalia R, Grewal S, Bisht D (2019). Effect of modified bass brushing technique and habitual brushing on the carriage of oral microbes in patients with fixed orthodontic appliances-A comparative study. Eur J Pharm Med Res.

[24] Walsh LJ, Healey DL (2019). Prevention and caries risk management in teenage and orthodontic patients. Aust Dent J.

[25] Saengphen T, Koontongkaew S, Utispan K. Reduction of dental biofilm cariogenicity in patients with fixed orthodontic appliances by a combined horizontal-charters-modified Bass brushing technique and dietary advice: a randomized clinical trial. (2022).

[26] An J-S, Lim B-S, Sug-Joon Ahn (2023). Managing oral biofilms to avoid enamel demineralization during fixed orthodontic treatment. Korean J Orthod.

[27] Sharma NC, Lyle DM, Qaqish JG, Galustians J, Schuller R (2008). Effect of a dental water jet with orthodontic tip on plaque and bleeding in adolescent patients with fixed orthodontic appliances. Am J Orthod Dentofac Orthop.

[28] Tyler D, Kang J, Goh HH. Effectiveness of Waterpik® for oral hygiene maintenance in orthodontic fixed appliance patients: a randomised controlled trial. J Orthodont 2023:14653125231173708. 10.1177/14653125231173708 PMC10693741 37203873

[29] Abdellatif H, Alnaeimi N, Alruwais H, Aldajan R, Hebbal MI (2021). Comparison between water flosser and regular floss in the efficacy of plaque removal in patients after single use. Saudi Dent J.

[30] Boyd R, Leggott P, Quinn R, Eakle W, Chambers D (1989). Periodontal implications of orthodontic treatment in adults with reduced or normal periodontal tissues versus those of adolescents. Am J Orthod Dentofac Orthop.

[31] Alstad S, Zachrisson BU (1979). Longitudinal study of periodontal condition associated with orthodontic treatment in adolescents. Am J Orthod.

[32] Freitas AO, Marquezan M, Nojima MDCG, Alviano DS, Maia LC (2014). The influence of orthodontic fixed appliances on the oral microbiota: a systematic review. Dent Press J Orthod.

[33] Kwon TH, Lamster IB, Levin L (2021). Curr Concepts Manage Periodontitis Int Dent J.

[34] Mancinelli-Lyle D (2023). Efficacy of water flossing on clinical parameters of inflammation and plaque: a 4‐week randomized controlled trial. Int J Dental Hygiene.

[35] Jahn CA (2010). The dental water jet: a historical review of the literature. Am Dent Hygienists’ Association.

[36] Goyal CR, Qaqish JG, Schuller R, Lyle DM (2018). Evaluation of the addition of a Water Flosser to Manual brushing on Gingival Health. J Clin Dent.

[37] Lang NP, Lindhe J (2015). Clinical periodontology and implant dentistry.

[38] Pretty I, Edgar W, Smith P, Higham S (2005). Quantification of dental plaque in the research environment. J Dent.

[39] Löe H, Silness J (1963). Periodontal disease in pregnancy I. Prevalence and severity. Acta Odontol Scand.

[40] Barnes CM, Russell CM, Reinhardt RA, Payne JB, Lyle DM (2005). Comparison of irrigation to floss as an adjunct to tooth brushing: effect on bleeding, gingivitis, and supragingival plaque. J Clin Dentistry.

[41] Preda C, Butera A, Pelle S (2021). The efficacy of powered oscillating heads vs. powered sonic action heads toothbrushes to maintain periodontal and peri-implant health: a narrative review. Int J Environ Res Public Health.

[42] Mwatha A, Olson M, Souza S (2017). Gingival health and plaque regrowth response following a four-week interdental hygiene intervention. J Clin Dentistry.

[43] Le Fouler A, Sylvie Jeanne S, Olivier Sorel O, Brezulier D (2021). How effective are three methods of teaching oral hygiene for adolescents undergoing orthodontic treatment? The MAHO protocol: an RCT comparing visual, auditory and kinesthetic methods. Trials.

[44] Bergkulla N, Hänninen H, Alanko O, Tuonmisto M, Miettinen A, Svedstrom-Oristo AL (2017). Introduction and assessment of orthognathic information clinic. Eur J Orthod.

[45] AlMoharib HS, AlAskar MH, AlShabib AN, Almadhoon HW, AlMohareb TS (2023). The effectiveness of dental water jet in reducing dental plaque and gingival bleeding in orthodontic patients: a systematic review and meta-analysis of randomized clinical trials [published online ahead of print, 2023 Sep 11]. Int J Dent Hyg.

[46] Ainamo J, Bay I (1975). Problems and proposals for recording gingivitis and plaque. Int Dent J.

[47] Löe H (1967). The Gingival Index, the Plaque Index and the Retention Index systems. J Periodontol.

[48] Xu X, Zhou Y, Liu C, Zhao L, Zhang L, Li H, Li Y, Cheng X. Effects of water flossing on gingival inflammation and supragingival plaque microbiota: a 12-week randomized controlled trial. Clin Oral Invest 2023. 10.1007/s00784-023-05081-4 PMC10212231 37231271

[49] Lyle DM, Qaqish JG, Goyal CR, Schuller R. Efficacy of the use of a water flosser in addition to an electric toothbrush on clinical signs of inflammation: 4-week randomized controlled trial. *Compend Contin Ed Dent* 2020; 41 (3): 170 2020, 177. 31904246

[50] Rosema N, Hennequin-Hoenderdos NL, Berchier CE, Slot DE, Lyle DM, van der Weijden GA (2011). The effect of different interdental cleaning devices on gingival bleeding. J Int Acad Periodontol.

[51] Worthington HV, MacDonald L, Pericic TP, Sambunjak D, Johnson TM, Imai P, Clarkson JE. Home use of interdental cleaning devices, in addition to toothbrushing, for preventing and controlling periodontal diseases and dental caries. Cochrane Database Syst Reviews 2019(4). 10.1002/14651858.CD012018.pub2 PMC6953268 30968949

[52] Sawan N, Ben Gassem A, Alkhayyal F, Albakri A, Al-Muhareb N, Alsagob E (2022). Effectiveness of Super Floss and Water Flosser in Plaque removal for patients undergoing Orthodontic Treatment: a Randomized Controlled Trial. Int J Dent.

[53] Caccianiga P, Nota A, Tecco S, Ceraulo S, Caccianiga G. Efficacy of home oral-Hygiene Protocols during Orthodontic Treatment with multibrackets and clear aligners: microbiological analysis with phase-contrast Microscope. Healthc (Basel) 2022, 10(11). 10.3390/healthcare10112255 PMC9691180 36360596

[54] Lyle DM. Relevance of the water flosser: 50 years of data. Compend Contin Educ Dent 2012, 33(4). 22536661

[55] Gorur A, Lyle DM, Schaudinn C, Costerton JW (2009). Biofilm removal with a dental water jet. Compendium.

[56] Ng E, Lim LP. An overview of different interdental cleaning aids and their effectiveness. Dent J (Basel) 2019, 7(2). 10.3390/dj7020056 PMC6630384 31159354

